# Endoscopic Duodenal Stent Placement for Malignant Gastric Outlet Obstruction Using the Balloon Anchoring Method

**DOI:** 10.1002/ccr3.70649

**Published:** 2025-07-17

**Authors:** Yuichi Takano, Naoki Tamai, Jun Noda, Masataka Yamawaki, Tetsushi Azami, Fumitaka Niiya, Fumiya Nishimoto, Naotaka Maruoka, Tatsuya Yamagami, Masatsugu Nagahama

**Affiliations:** ^1^ Division of Gastroenterology, Department of Internal Medicine Showa University Fujigaoka Hospital Yokohama Kanagawa Japan

**Keywords:** balloon anchoring method, endoscopic duodenal stent placement, gastric outlet obstruction, pancreatic cancer

## Abstract

Endoscopic duodenal stent placement, a widely used procedure for managing malignant gastric outlet obstruction, has a high technical and clinical success rate. However, precise confirmation of stricture site during the procedure is sometimes challenging. This case report introduces a novel balloon anchoring method to address this challenge. An 87‐year‐old female diagnosed with gastric outlet obstruction due to pancreatic cancer underwent endoscopic duodenal stent placement. However, the stricture site was unclear on fluoroscopic imaging. A balloon catheter was inserted beyond the stricture, inflated, and retracted toward the oral side. The balloon was anchored on the anal side of the stricture, facilitating precise stricture site confirmation. Subsequently, an uncovered duodenal metal stent was successfully placed. The procedure was completed without adverse events, and the patient resumed eating the following day. By using the balloon anchoring method, duodenal stricture can be easily and accurately evaluated. After confirming the stricture, a duodenal stent can be placed at the optimal position. We believe this technique will contribute to the safety and reliability of the procedure.

## 1 Introduction

Malignant gastric outlet obstruction (GOO) is caused by malignant neoplasms, with gastric and pancreatic cancers as the main causes [1]. Endoscopic treatment approaches for malignant GOO include duodenal stent placement and endoscopic ultrasound‐guided gastroenterostomy (EUS‐GE) [2, 3, 4, 5, 6, 7, 8]. A recent meta‐analysis of malignant GOO revealed that the technical success rate was slightly better with duodenal stent placement than with EUS‐GE (99.4% vs. 95.3%), whereas duodenal stent placement and EUS‐GE had comparable rates of clinical success (88.9% and 89.0%, respectively) and overall procedural adverse events (18.7% and 21.9%, respectively) [9]. The rates of malignant GOO recurrence and reintervention were lower with EUS‐GE than with duodenal stent placement.

In endoscopic duodenal stent placement, accurate confirmation of the stricture site is essential for procedural success. However, precise confirmation of the stricture site during the procedure is sometimes challenging. This case report introduces a novel balloon anchoring method to address this challenge.

## 2 Case Report

An 87‐year‐old female diagnosed with unresectable pancreatic head (uncus) cancer was transferred to our hospital due to persistent postprandial vomiting lasting for 2 days. Her past history includes bronchial asthma and chronic heart failure. She had undergone ureteral stent placement a month earlier for left ureteral obstruction caused by cancerous peritonitis. Her vital signs were a body temperature of 36.6°C, blood pressure of 105/64 mmHg, and heart rate of 91/min. Physical examination revealed mild epigastric tenderness, but no muscular guarding or rebound pain.

### 2.1 Diagnosis, Investigations, and Treatment

Plain abdominal computed tomography (CT) revealed a malignant GOO due to pancreatic cancer. Endoscopic duodenal stent placement was planned.

**FIGURE 1:**
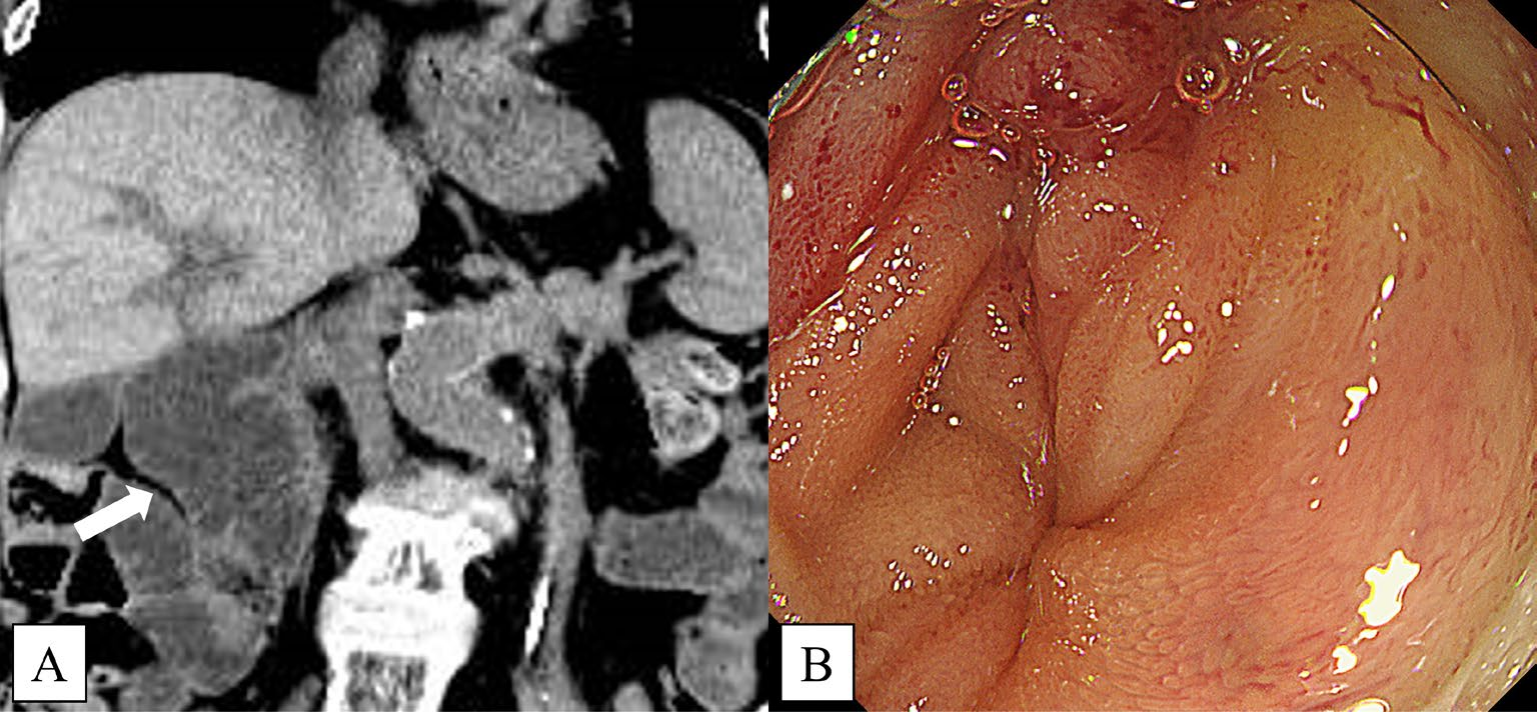
(A) Abdominal computed tomography revealed a malignant gastric outlet obstruction due to pancreatic head cancer. The dilated duodenum can be seen (arrow). (B) A severe stricture was observed in the horizontal portion of the duodenum.

**FIGURE 2:**
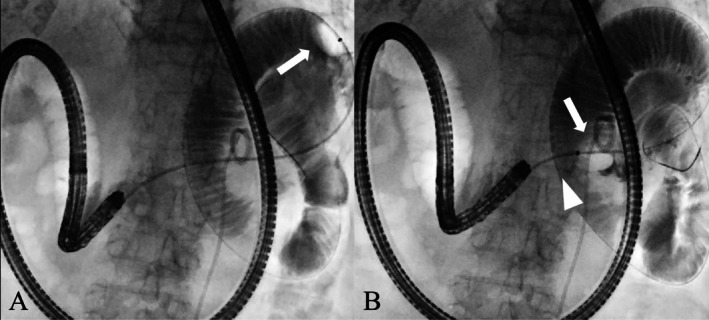
(A) A balloon catheter (Extractor ProXL, Boston Scientific, Tokyo, Japan) was inserted beyond the stricture, inflated (arrow), and retracted toward the oral side. (B) The balloon was anchored on the anal side of the stricture (arrow), facilitating precise stricture site confirmation (arrowhead).

**FIGURE 3:**
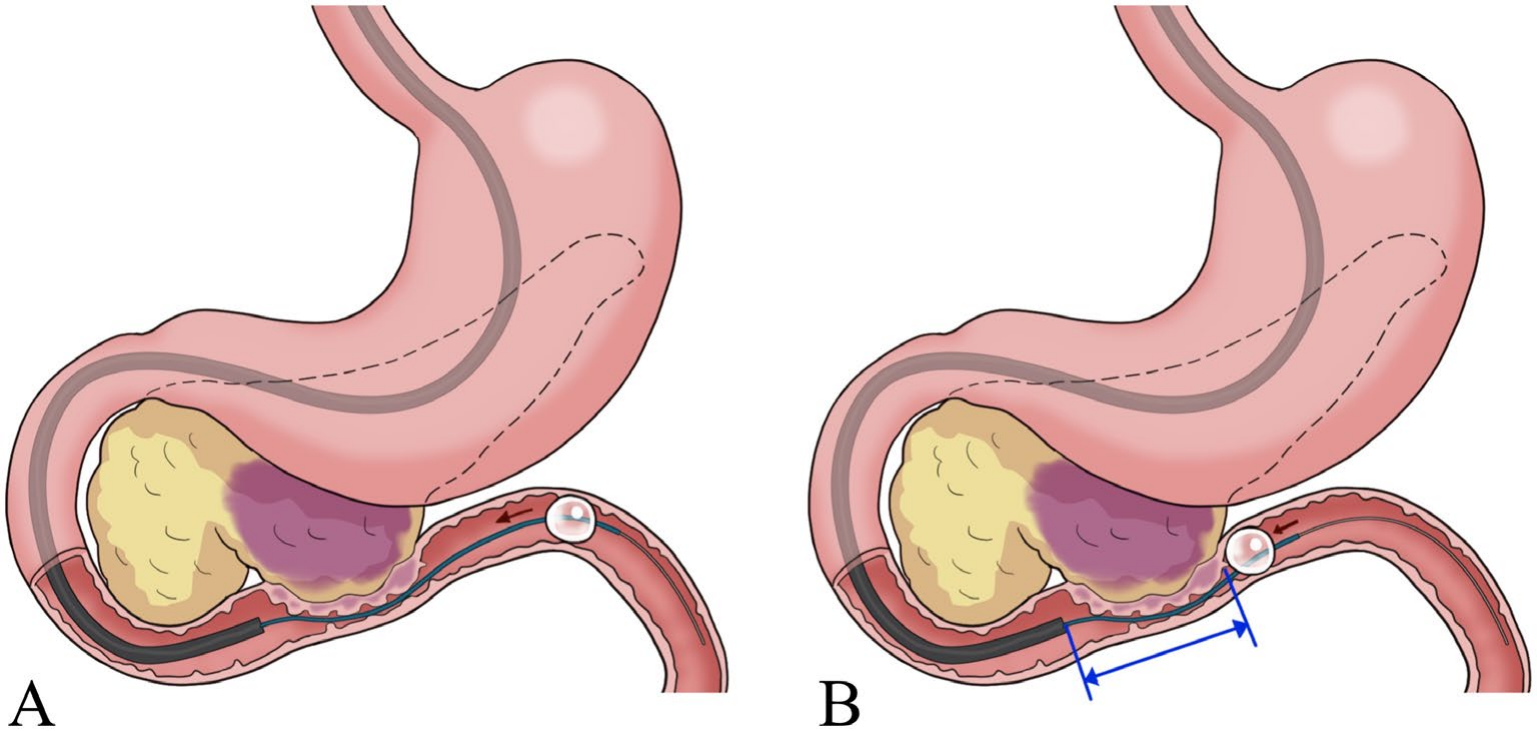
A schema of the balloon anchoring method. (A) A balloon catheter was inserted beyond the stricture, inflated, and retracted toward the oral side. (B) The balloon was anchored on the anal side of the stricture, facilitating precise stricture site confirmation.

**FIGURE 4:**
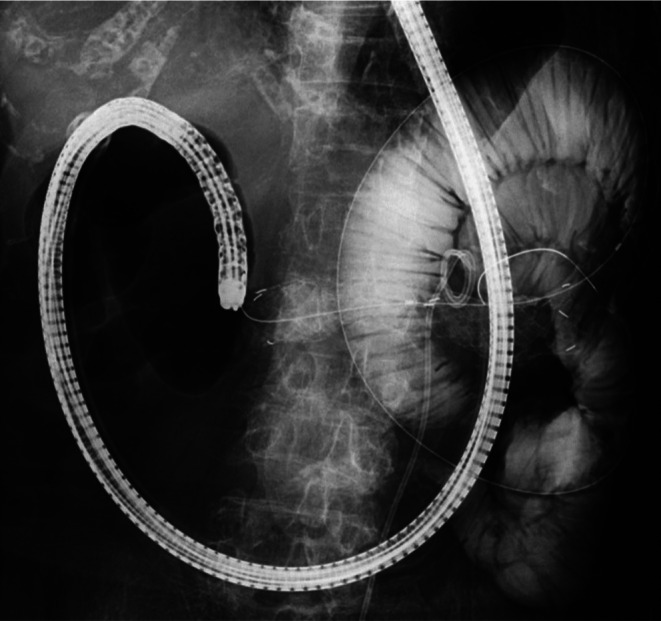
An uncovered metal stent was successfully placed across the stricture.

After inserting a short‐type single‐balloon enteroscope (SIF‐H290S, Olympus, Tokyo, Japan), a severe stricture was found in the horizontal portion of the duodenum that impeded scope passage (Figure 1). A 0.025‐in. guidewire (Visiglide2, Olympus) breached the stricture, and a contrast medium was injected through a catheter. However, the stricture site was unclear due to peristalsis and overlap of the small intestine. A balloon catheter (Extractor ProXL, Boston Scientific, Tokyo, Japan) was inserted beyond the stricture, inflated, and retracted toward the oral side. The balloon was anchored on the anal side of the stricture, facilitating precise stricture site confirmation (Figures 2 and 3). Subsequently, an uncovered metal stent (Niti‐S Pyloric/Duodenal Stent, Century Medical, Tokyo, Japan), 22 mm in diameter and 12 cm in length, was successfully placed across the stricture (Figure 4).

### 2.2 Outcome and Follow‐Up

The procedure was completed without adverse events, and the patient resumed eating the following day.

## 3 Discussion

The conventional treatment option for malignant GOO is surgical gastrojejunal bypass, although endoscopic treatment has been widely adopted as a less invasive approach in recent years. Endoscopic treatment options for malignant GOO include duodenal stent placement and EUS‐GE. The most important advantage of endoscopic duodenal stent placement is its ability to expand the physiological digestive route, which prevents the leakage of digestive fluids into the abdominal cavity [2, 3, 4, 5, 6, 7, 8, 9].

In patients with malignant GOO, the presence of bile duct obstruction should be evaluated. Malignant GOO caused by pancreatic cancer may simultaneously cause bile duct obstruction, which requires biliary drainage as well. In patients with duodenal stenosis across Vater's ampulla, endoscopic transpapillary biliary drainage is often difficult, and EUS‐guided or percutaneous biliary drainage should be considered [10].

During endoscopic duodenal stent placement, a guidewire is used to break through the stenosis, a catheter is inserted deep into the stenosis, and the length of the stenosis is confirmed with contrast injection. The stent can be stabilized by positioning the center of the stent at the stenosis site. Confirmation of the stricture site is essential for procedural success.

Evaluation with computed tomography is important to predict the stricture site before the procedure, whereas gastrointestinal imaging should be performed to confirm the stricture site during the procedure. However, confirmation of the stricture may be difficult due to overlap and peristalsis of the small intestine, and misrecognition of the stricture site can result in stent misplacement or migration.

By using the balloon anchoring method, duodenal stricture can be easily and accurately evaluated. The balloon is inserted beyond the stricture, inflated, and pulled back toward the oral side, thereby anchoring the balloon on the anal side of the stricture. After confirming the stricture, a duodenal stent can be placed at the optimal position.

## 4 Conclusions

By using the balloon anchoring method, duodenal stricture can be easily and accurately evaluated. We believe this technique will contribute to the safety and reliability of the procedure.

## Author Contributions

**Yuichi Takano:** conceptualization, data curation, formal analysis, writing – original draft, writing – review and editing. **Naoki Tamai:** formal analysis. **Jun Noda:** formal analysis. **Masataka Yamawaki:** formal analysis. **Tetsushi Azami:** formal analysis. **Fumitaka Niiya:** formal analysis. **Fumiya Nishimoto:** formal analysis. **Naotaka Maruoka:** formal analysis. **Tatsuya Yamagami:** formal analysis. **Masatsugu Nagahama:** formal analysis.

## Consent

Written informed consent was obtained from the patient's parents to publish this report in accordance with the journal's patient consent policy.

## Conflicts of Interest

The authors declare no conflicts of interest.

## Data Availability

The data that support the findings of this study are available from the corresponding author upon reasonable request.
